# Stress and substance use disorders: risk, relapse, and treatment outcomes

**DOI:** 10.1172/JCI172883

**Published:** 2024-08-15

**Authors:** Rajita Sinha

**Affiliations:** The Yale Stress Center, Yale School of Medicine, Department of Psychiatry, New Haven, Connecticut, USA.

## Abstract

Stress has long been associated with substance misuse and substance use disorders (SUDs). The past two decades have seen a surge in research aimed at understanding the underlying mechanisms driving this association. This Review introduces a multilevel “adaptive stress response” framework, encompassing a stress baseline, acute reaction, and recovery with return-to-homeostasis phase that occurs at varying response times and across domains of analysis. It also discusses evidence showing the disruption of this adaptive stress response in the context of chronic and repeated stressors, trauma, adverse social and drug-related environments, as well as with acute and chronic drug misuse and with drug withdrawal and abstinence sequelae. Subjective, cognitive, peripheral, and neurobiological disruptions in the adaptive stress response phases and their link to inflexible, maladaptive coping; increased craving; relapse risk; and maintenance of drug intake are also presented. Finally, the prevention and treatment implications of targeting this “stress pathophysiology of addiction” are discussed, along with specific aspects that may be targeted in intervention development to rescue stress-related alterations in drug motivation and to improve SUD treatment outcomes.

## Introduction

Stress is a common reason for drug use and misuse in the US and the Western world and is evoked frequently in linking stress and substance use disorders (SUDs) (1, 2). Psychological theories view drug misuse as a coping mechanism to reduce stress, anxiety, tension, withdrawal, and abstinence-related distress, and as a means for self-medication (1, 3, 4). Misuse as a coping mechanism has also been identified as a way of understanding the frequent co-occurrence of other stress-related psychiatric illnesses, such as mood and anxiety disorders and post-traumatic stress (PTSD), with SUDs (5). Neurobiological models highlight how drug-related neuroadaptations in the circuits underlying limbic emotional states, reinforcement learning, self-control, and decision-making contribute to drug-related distress states such as withdrawal and abstinence, which in turn promote drug seeking (2, 6). Additionally, the incentive-sensitization model of addiction highlights neuroadaptations pertaining to incentive salience that may drive the escalation of drug use (7, 8); this model has been extended to explain stress-related sensitization of drug salience, which promotes increases in craving and drug use escalation, thereby affecting SUD risk and the severity and course of SUD (9–12). However, specifically how these processes are engaged during stress, trauma, and adversity and the psychobiological stress responses that may drive addiction need further explication to identify the best ways to target them for addiction prevention, treatment, and recovery.

First, this Review introduces an “adaptive stress response” conceptual framework to identify operational components of the multilevel stress responses that occurs based on the intensity, controllability, predictability, and chronicity of the stressful event. This adaptive stress response is susceptible to alterations and disruptions on the basis of these aspects, which in turn influences maladaptive coping. Next, the Review outlines three broad sets of environmental and individual-level stressors associated with SUD risk, relapse, and treatment failure. The specific subjective, peripheral, and neurobiological disruptions characterized as the “stress pathophysiology of addiction” are described, and their association with future drug use escalation, maintenance, and relapse risk in SUD is also presented. Finally, specific aspects of this stress pathophysiology of addiction that may serve as potential targets for prevention and treatment are discussed, with research examples showing that restoration of the multilevel adaptive stress response is associated with improvements in SUD treatment outcomes.

## Conceptualization of the multilevel adaptive stress response

**Figure 1:**
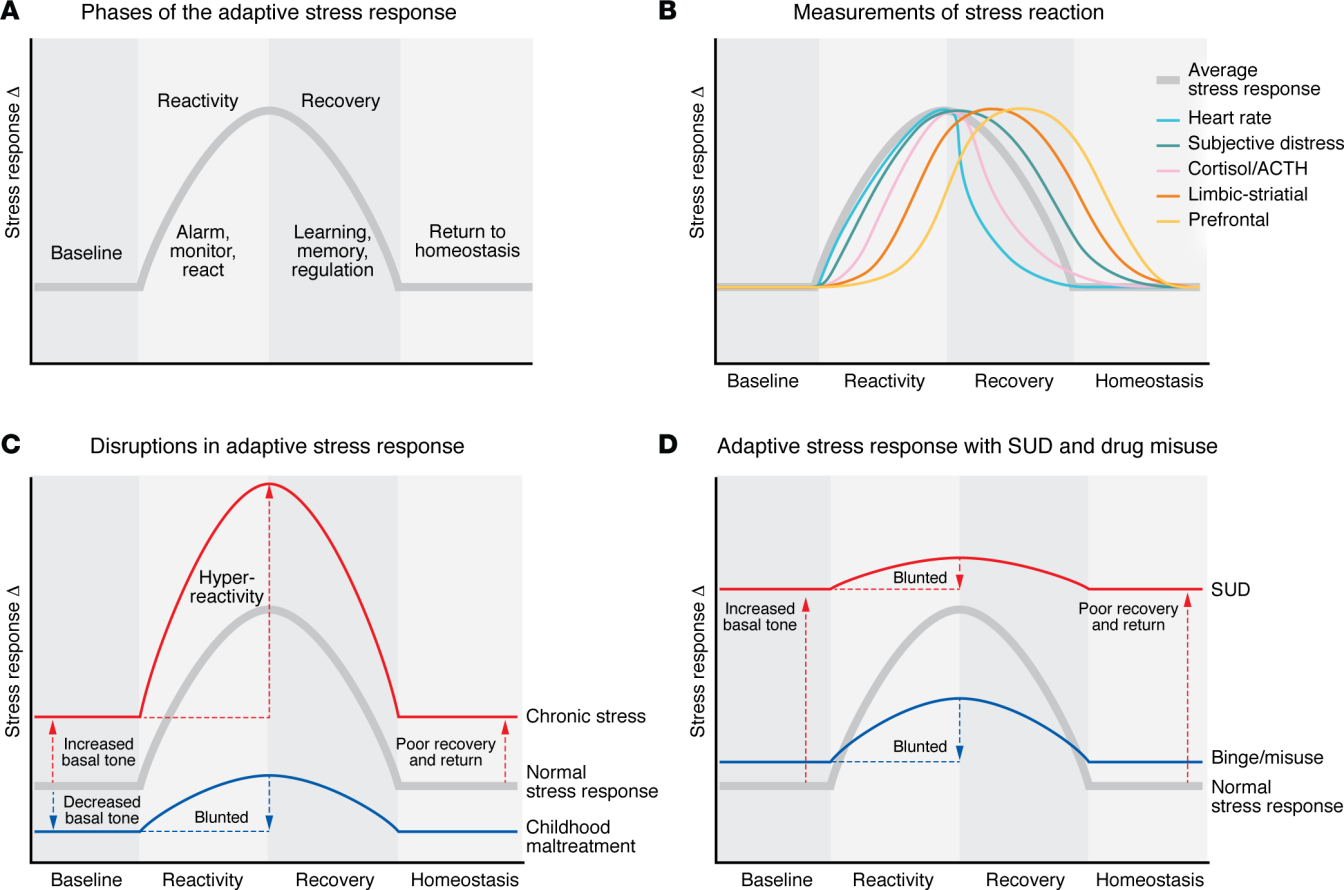
Conceptual schematic of the multilevel adaptive stress response across domains. (**A**) The stress response in three phases, including *baseline* non-stress, relaxed state; the *stress reaction* state, including alerting, alarm, and immediate response if needed; and the recovery or regulatory state, including *recovery and return to homeostasis*. (**B**) Variation in stress reaction across levels of measurement and timescales based on intensity, sustained/repeated exposures, controllability, and predictability. (**C**) Based on research evidence, a schematic of the disruptions in the adaptive stress response phases with chronic repeated stress and with early-life stress/childhood maltreatment. (**D**) The documented changes across phases with binge and escalated drug use and in SUD.

Humans are uniquely wired to face and respond to challenges and overwhelming situations. Referred to as “stress” or “stressors,” these have been defined as any highly challenging, threatening, or overwhelming internal (e.g., physical such as pain, hunger, sleep deprivation) or external psychosocial events or series of events that result in adaptive and maladaptive processes required to regain homeostasis and/or stability (9, 13). This dynamic and adaptive stress response may be represented as an inverted-U function, similar to the Yerkes-Dodson inverted-U function describing the effects of stress arousal on performance (ref. 14; cf. ref. 15), with three specific phases: (i) the baseline non-stress state; (ii) the reaction function, encompassing the internal alarm system, serving to alert or signal threat, danger, or challenge, and inclusive of the multilevel stress responses that occur under high-stress states to avert or cope with the challenge; and (iii) the recovery phase, encompassing regulatory and adaptive processes required to facilitate a return to homeostasis. The reaction and regulatory phases include learning, memory, and self-control processes that support survival, longevity, and adaptive coping for facing future stressful events (see Figure 1A).

Basic science and human neuroimaging research over the last two decades has supported the occurrence of an acute and adaptive stress response that engages multilevel sensory, physiologic, interoceptive, behavioral, emotional, cognitive, and metacognitive brain networks in a parallel, distributed, and dynamic manner; to achieve optimally flexible and adaptive responses across multiple levels of functioning (16–21). For example, growing evidence supports a role for an acute cortisol response in cognitive and emotional regulation of the stress response, particularly in its interaction with prefrontal neural circuits (22, 23). The temporal aspects of the glucocorticoid response to acute stress (24), especially the delayed cortisol response, have been related to prefrontal activation during stress and linked to cognitive and emotional regulation in stress recovery (25). Furthermore, the acute coordinated multilevel subjective, physiological, and striatal dopaminergic response to acute stress has been documented using PET imaging (26). This multilevel acute stress response is dysregulated in those with chronic adversity, with a blunting of the physiologic and dopaminergic activation in response to acute stress and greater subjective distress (26). The extent to which the stress is (i) uncontrollable, (ii) unpredictable, (iii) highly intense, and (iv) relentless (repeated, chronic, or long) drastically shapes the engagement of multiple psychobiological responses across domains (autonomic, endocrine, neural, cognitive, subjective, immune, metabolic, cellular, molecular, genomic) (27–31) to achieve effective, flexible regulation and recovery and a return to homeostasis (13, 16, 32) (see Figure 1B).

The multilevel stress reaction may vary in intensity and rapidity of response across domains and serves to alert and signal challenge and mobilize processes to respond to the stress. Simultaneously, the stress reaction encodes salience and value aspects of stress stimuli cues to engage learning and memory processes, as well as prefrontal decision-making and regulatory circuits that execute initial immediate survival behaviors if needed. In addition, the stress reaction activates cognitive, emotional, and social behavioral processes to initiate stress regulation and serve long-term adaptation and stress resilience (11, 16, 17, 29). For example, a stressor such as being chased by a dangerous animal mobilizes immediate sensory processing inclusive of primary and sensory association cortices and may facilitate sensory-motor networks for fast, automatic biological and behavioral responding (e.g., running). In parallel, this stressor invokes the well-known stress biological responses encompassing the peripheral autonomic nervous system (ANS), stimulating sympathetic arousal, and the hypothalamic-pituitary-adrenal (HPA) axis to elicit peripheral cortisol activation (32, 33, 34). Further, central interoceptive and negative feedback signaling via the insula, amygdala, hippocampus, and medial prefrontal cortical networks engages cortico-limbic-striatal learning and motivation systems necessary for learning, adaptation, and regaining cognitive control (17, 19, 20, 22, 23, 25). Depending on the nature of the stressor (physical, cognitive) and duration of stress exposure, there could be a need to mobilize energy and acute inflammation toward host defense and physical survival processes (30, 31, 34). Thus, the stress reaction may include rapid increases in heart rate, blood pressure, and other autonomic metrics and may be combined with secondary neurobehavioral processing of sensory cues to mobilize an individual toward action. Simultaneously, the more gradually responding HPA axis is stimulated, beginning with the release of corticotropin-releasing factor (CRF) and eventually ending with the release of cortisol into the body (22, 25, 35, 36, 37). Glucocorticoid-mediated and direct mobilization of metabolic responses may coordinate access to additional energy sources in the body from stored protein and fat to elicit action while mobilizing immune responses that facilitate host defense systems against challenges such as infection. Thus, the individual domains’ responses may occur along varying timescales based on stress intensity, response demand, and physiology, as noted in previous work (33, 34).

**Figure 2:**
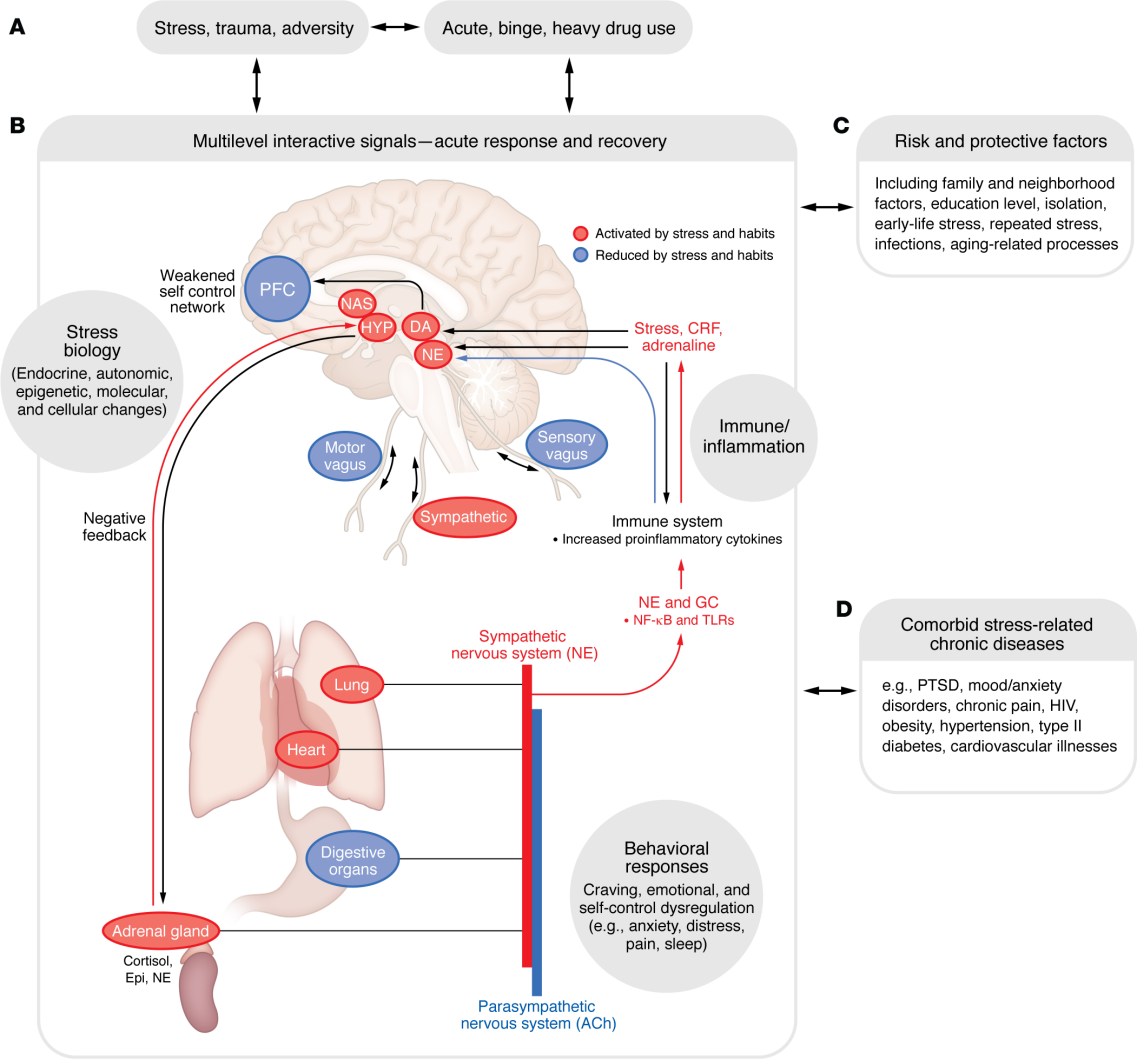
Pathways and processes involved in the multilevel stress response. A heuristic model shows that high, repeated, and chronic stress and traumatic events as well as binge and heavy drug misuse (**A**) target the interactive parallel multilevel neural, behavioral, immune, endocrine, and molecular responses to coordinate both the acute adaptive stress response and the regulatory processes for recovery and return to homeostasis. (**B**) This multilevel stress response system functions as the substate for emergent disruptions across neurobiological pathways as well as behavioral symptoms under pathophysiological conditions; and is further influenced by risk and protective factors (**C**). Changes and disruptions may occur at different levels based on individual vulnerabilities, thereby increasing risk of specific additional stress-related illnesses often comorbid with SUD (**D**). ACh, acetylcholine; DA, dopamine; Epi, epinephrine; GC, glucocorticoid; HYP, hypothalamus; NE, norepinephrine; PFC, prefrontal cortex.

In addition to such biological arousal, there are parallel higher-order signals via interoceptive, physiological, cognitive, emotional, and behavioral responses that initiate stress coping, as documented in several previous models describing multilevel stress coping responses (1, 13, 16, 17, 19, 28, 38). Individuals change their understanding of the stressor, decision-making patterns, emotions, and behavioral responses by engaging or disengaging prefrontal networks that underlie coping (16–19, 39). Coping may also take the form of stress soothing, support, avoidance, and seeking adaptive rewarding behaviors through social connections that contribute to stress recovery and return to homeostasis (39). Social neuroscience research has further identified cortico-limbic-striatal networks involved in such social coping, illustrating an additional important coping substrate available for promoting adaptive stress responses (24, 39–42). Thus, the stress response is an adaptive, dynamic, flexible, and indispensable facet of the individual wherein the multilevel responses interface with the social-contextual milieu to effectively exercise personal and social agency and control. The description and examples cited above illustrate the conceptual framework of the adaptive stress response and its highly complex, dynamic, and interactive nature. Given the complexity, this Review concerning stress and addiction is primarily focused on the physiological, endocrine, neural, subjective/cognitive, and behavioral domains; a detailed discussion of metabolic and immune aspects of the association, for which there is less evidence, is beyond the scope of the Review. Figure 2 presents a schematic of this multilevel adaptive stress response across domains and additional risk and protective factors and stress-related illnesses that are frequently comorbid with SUD.

The next section describes three specific types of high, repeated, and chronic levels of stress that can overwhelm the exquisitely wired adaptive stress response system with substantial, sustained disruption of the dynamic flexible responses. This disruption can change the basal subjective state and physiologic tone, the phasic stress response, as well as the stress recovery phase without a return to homeostasis, as discussed in previous psychosocial and psychobiological stress adaptation models (43, 44) and illustrated in Figure 1, C and D.

## Factors affecting stress response, learning, and motivation

### Stress, trauma, and adversity effects on addiction risk

Mounting evidence from population-based and clinical studies indicates statistically significant associations between social adversity, child and adult traumas, and uncontrollable and unpredictable stressful events and addiction risk (45–53). The broad categories of stressors and adverse life events linked to addiction risk are listed in Table 1. For example, research from the CDC-Kaiser ACE Study showed that individuals with a greater number of adverse childhood experiences (ACEs) are more prone to develop alcohol use disorder (AUD) and SUDs (52, 53). Recent evidence from a number of large-scale, longitudinal studies of children and adolescents have shown that greater social adversity and more numbers of stressful life experiences increase the likelihood of initiating drug intake and at earlier ages, as do drug-associated family environments, e.g., in which parents use substances, including in the context of prenatal exposure (54–58).

Notably, traumatic and repeated or chronic adverse life events during early life or in adolescence may result in dysregulation of the multilevel stress responses shown in Figure 1C. Chronic and repeated psychosocial adversity is associated with chronic activation of the HPA axis and pervasive sensitization of subjective distress and dysregulation of neurobiological responses (58–61). Early childhood trauma and maltreatment are associated with profound alterations in autonomic responses, as measured by heart rate, heart rate variability, and blood pressure responses (62–66), flattening of the diurnal cortisol response, and blunted cortisol and cardiovascular reactivity to a laboratory stressor (60–64). Such alterations in the HPA axis responses (49, 52, 58, 64, 67–72) and in the autonomic responses (54, 55, 65, 66, 73–75) have each been associated with increased addiction risk. While the severity, persistence, and psychosocial context of the maltreatment and/or adversity are important variables in the specific manifestation of the stress disruption (76) (as modeled in Figure 1C), the wealth of evidence clearly links sustained disruption of the adaptive stress responses with specific associations to risk of future substance use and misuse and related psychiatric and medical comorbidities, as illustrated in Figure 2.

#### CNS response to stress and risk of SUDs.

Neuroimaging studies of trauma, adversity, and chronic stress, as well as prenatal drug exposure, have documented lasting changes in the structure, function, and regulation of the prefrontal cortical, limbic, and striatal brain networks involved in processing distress, emotions, reward, and higher cognitive or executive control functions (see refs. 9, 20, 52 for review). For example, structural MRI (sMRI) studies of the human brain have shown that psychosocial adversity, childhood maltreatment, adult trauma, and recent life stressors such as those listed in Table 1 are associated with lower gray matter volume in critical limbic, striatal, and prefrontal cortex regions involved in stress and reward processing, stress coping, and regulation and cognitive control (77–82). The specific areas include the orbitofrontal cortex (OFC), ventromedial prefrontal cortex (VmPFC), dorsolateral and dorsomedial prefrontal cortex (DLPFC and DMPFC), amygdala, hippocampus, and insula regions of the brain; and volume changes in these regions are associated with an increased likelihood of substance use initiation or drug escalation (54, 55, 83). Consistent with these associations of stress with gray matter volume, functional neuroimaging research has also shown that stress exposure is associated with lower medial and dorsolateral prefrontal function and greater limbic-striatal activation — as measured by functional MRI (fMRI) (17, 29, 78, 81) — a brain pattern associated with low behavioral and cognitive control over stress and reward (84–86). Importantly, a key substrate of the link between stress and addiction risk is disrupted and blunted peripheral interoceptive feedback and central stress activity, which alter striatal motivational reward circuits, increasing susceptibility to addiction.

### Drug misuse effects on stress responses and regulation

Psychoactive drugs directly affect the adaptive stress response (depicted in Figure 1, A and B), powerfully activating or blunting the peripheral autonomic and HPA axis stress responses as well as affecting central, metabolic, and immune responses and modulating cognitive, emotional, and behavioral effects (refs. 9, 10; see ref. 12 for review). For example, acute administration of moderate to high doses of nicotine (87), cannabis (88), alcohol (89, 90, 91), or cocaine (92, 93) activates the autonomic, HPA, and noradrenergic stress arousal pathways (see ref. 12 for review). Most psychoactive substances, except opioids and benzodiazepines, also stimulate catecholamine release, which with chronic exposure can induce tachycardia and hypertension (10, 12). In both laboratory and real-world studies, acute alcohol consumption reduces parasympathetic tone and increases sympathetic arousal during sleep in individuals without AUD or SUD (97, 98).

While most substances acutely stimulate the HPA axis and autonomic responses, these peripheral physiological responses to substances become less-reactive and blunted with repeated and escalating use, as with drug tolerance responses (12). Furthermore, there are basal or tonic state shifts, wherein HPA axis activity may become chronically elevated. This effect has been documented with nicotine, alcohol (44, 89, 99), cocaine, and cannabis (88, 100, 101). Blunted phasic responses in cortisol reactivity akin to tolerance have been documented in binge and heavy use of cannabis, nicotine, alcohol, and opiates (49, 88, 101, 102), as have blunted stress-related cytokine responses (103, 104). Chronic and heavy alcohol and substance use can also alter autonomic processes, with long-term effects including reduced heartbeat complexity, impaired vagal function, and lower parasympathetic activity (12). In individuals with heavy alcohol use, there is dampened parasympathetic tone during sleep (105), as well as reduced resting heart rate variability (HRV) and increased reactive high-frequency HRV, which are associated with enhanced craving and relapse vulnerability (106). More importantly, the alterations in stress- and drug-related arousal and increased subjective stress have also been associated with increased drug craving and intake (89, 90, 102, 107–113). These findings suggest that disruptions in peripheral stress biology are a potential risk marker for the progression from binge and heavy drug intake to risk of SUD, and represent changes that may be targeted for intervention development (52, 107, 114).

#### Neural responses to binge and heavy drug use.

Binge and heavy substance use also result in neurobiological alterations in stress and reward circuits that further promote drug motivation, craving, and escalated drug intake. Multiple studies have shown lower structural gray matter volume and disrupted drug- and stress-induced functional responses in corticolimbic striatal regions of the amygdala, nucleus accumbens, OFC, hippocampus, and insula, as well as multiple prefrontal regions, including the VmPFC, DLPFC, and DMPFC, in binge and heavy users of substances such as nicotine (115, 116), alcohol (99, 117–121), cocaine (122), methamphetamine (123), and heroin (124, 125) compared with controls (also see ref. 126 for review). Importantly, the peripheral disruptions described have also been associated with altered subjective emotional responses to stress and drug and changes in the striatal and prefrontal regions, suggesting the presence of changes in interoceptive circuits across levels of the stress response that may contribute to increased drug craving and intake (99, 127, 128). Thus, with binge and heavy drug use, there are significant changes in neural circuits involved in stress reactivity and motivation, as well as in stress-regulatory regions, underlying adaptive choices, decision making, self-control, and coping. A schematic of the representative disruption in the phasic peripheral and neurobiological stress response with a progression of hyperactive basal (tonic) and altered homeostasis that builds with increasing chronic and heavy drug misuse is illustrated in Figure 1D.

### Stress responses and outcomes during withdrawal and abstinence

Repeated abstinence and withdrawal from chronic, binge drug intake is associated with a well-documented subjective distress state marked by negative emotions, such as anxiety, depressed mood, pain, fatigue, sleep difficulties, and other physical symptoms specific to the type of drug withdrawal (i.e., alcohol or opiates) with additional symptoms of tremor, nausea, agitation and aggression, high basal autonomic tone (basal heart rate and blood pressure) (129–135). While medical detoxification for alcohol and opiate dependence reduces physical symptoms (129, 132, 133), the heightened distress state and associated dysregulation in stress biology also occur during abstinence from cocaine, cannabis, and nicotine, and the negative emotional state, anxiety, and altered stress biology affect compulsive drug motivation and risk of relapse and treatment failure (6, 134, 136, 137). Higher levels of childhood trauma and maltreatment may exacerbate these abstinence symptoms and augment the risk of relapse and treatment failure (137, 138, 139). Notably, states of abstinence and withdrawal from nicotine, alcohol, opiates, cocaine, and cannabis are associated with blunted adrenocorticotropic hormone (ACTH), cortisol (140, 141, 142–148), and cytokine responses (150) to stress and to CRF administration (149). Furthermore, increased basal HPA axis markers and autonomic arousal (heart rate, HRV) have been reported in smokers and individuals with AUD (146–148, 151).

Research has also shown that the disrupted patterns of the multilevel stress response are predictive of future risk of relapse and treatment failure. Stress exposure in individuals with SUD is associated with high levels of drug craving, as with drug cue reactivity; enhanced negative mood and anxiety; high basal and blunted phasic autonomic and HPA axis responses; disrupted HRV responses; and increased relapse risk and greater drug intake in individuals with AUD (145, 151–153), nicotine use disorder (148, 154, 155), and cocaine use disorder (112, 156, 157, 158).

#### CNS response in drug motivation and relapse risk.

Multiple fMRI, PET, and sMRI neuroimaging studies have shown disrupted limbic-striatal and prefrontal circuits involved in stress-, drug-, and drug cue–related activity that predict an increase in drug craving, drug intake, and relapse risk (86, 159). For example, hyperactivity in the limbic-striatal regions is associated with elevated levels of emotional distress and heightened drug craving (29, 160–166). Furthermore, activation patterns in the VmPFC, DLPFC, ventral striatum, and insula networks during stress and drug-cue states and in early abstinence have been documented in individuals with SUD when compared with healthy controls and in association with relapse and treatment outcomes (161–168). Studies have shown that disruptions in executive control and incentive salience networks involved in regulating stress- and cue-related drug craving and stress responses predict drug craving, relapse, and treatment outcomes in SUD (167), and there is some evidence of recovery in these circuits with abstinence (169, 170). Recent PET studies have shown lower endogenous dopamine or lower availability of dopamine receptors (171–175) and lower cannabinoid receptor binding (176) under acute stress or with chronic drug use; moreover, altered dopamine receptor binding (171–175), higher stress-related κ opioid receptor availability (171, 177), and higher cortisol-regenerating enzyme availability (178) in chronic drug misuse have been associated with greater probability of engaging in drug use, greater amount of drug intake, and greater risk of adverse outcomes in SUD.

**Figure 3:**
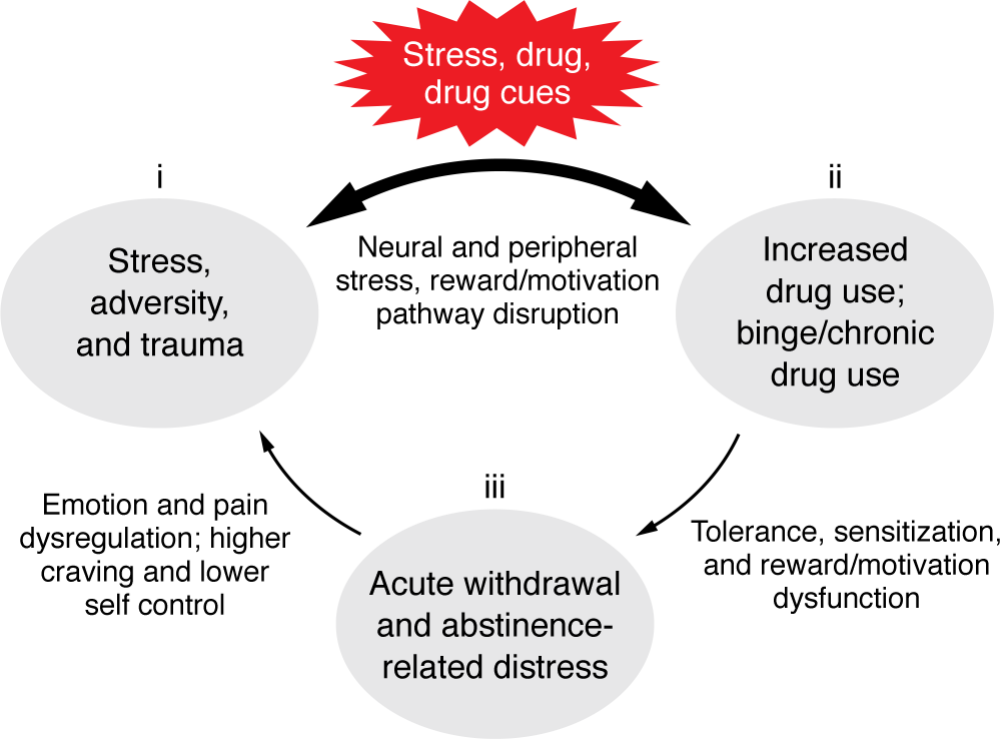
Maladaptive alterations to the adaptive stress response. Model showing the interactive effects of (i) stress, trauma and adversity, (ii) increased drug use, binge/chronic use, and (iii) acute withdrawal and abstinence-related distress,as the three stress factors presented in the section entitled “Factors affecting stress response, learning, and motivation.” With increasing cumulative aggregation of each of these factors, the natural adaptive processes involved become altered, which results in greater multilevel disruptions in stress, reward, and motivation pathways. Drug-related processes of tolerance, sensitization and withdrawal further facilitate the *feed-forward* disruptions in emotion, pain, and reward pathways to promote increased craving and risk of drug use escalation, relapse, and treatment failure.

sMRI studies have also shown greater atrophy in stress-regulatory regions of the bilateral OFC, the right medial PFC, and anterior cingulate cortex (ACC) in individuals with SUD and AUD who relapsed compared with those who remained abstinent and healthy controls (179, 180). In addition, large-scale sMRI studies have documented significant gray matter atrophy in the ACC, insula, OFC, and other prefrontal regions involved in stress regulation in individuals with SUD relative to controls (79, 181–183). Together, these findings indicate that chronic drug misuse with repeated bouts of withdrawal and abstinence results in considerable disruptions in stress circuits involved in adaptive stress responses. These disruptions occur in conjunction with the subjective distress state as well as the peripheral stress biological disruptions described above. Figure 1D presents a schematic of this disrupted neurobiological state marked by heightened basal tone and blunted phasic stress responses and dysfunctional regulatory mechanisms that prevent adaptive recovery and return to homeostasis. Such a disrupted maladaptive stress response exerts greater allostatic load, which is purported to drive increased drug craving and compulsive intake, as postulated in a number of integrated reviews on stress and addiction (2, 6, 10, 44). It is this underlying stress pathophysiology that occurs across multiple stress response domains in a feed-forward manner that is associated with greater risk of treatment failure in SUD (see Figure 3).

## The stress–drug use cycle and treatment failure

There are several key takeaways from the findings of neurobiological adaptations to the adaptive stress response encompassing parallel learning, memory, and regulatory pathways (shown in Figure 1, A and B) and disrupted by chronic stress, trauma and cumulative adversity, binge and heavy drug use, and repeated bouts of withdrawal and abstinence (Figure 1, C and D). The extent of stress- and drug misuse–related changes may vary as a function of genetic vulnerability (184) — though a discussion of this topic is beyond the scope of this Review — and demographic and experience-related risks and protective factors (185–188) (highlighted in Figure 2) known to impact addiction pathophysiology (1, 3, 9). The extent of neural and psychobiological manifestations of stress disruptions may vary based on cumulative stress load and the extent of drug misuse and SUD severity (e.g., specific drug effects, drug use amounts, frequency and recency of use, repeated withdrawals); these in turn can affect the psychological symptoms associated with SUD, such as high subjective distress, acute and sensitized stress, pain and cue reactivity, increased craving, impulsive responding, anxiety, increased negative mood, sleep difficulties, pain symptoms, and other psychological and medical morbidities (5, 9, 10, 188, 189).

Research also suggests that the accumulation of the stress–drug use severity risk, which is the collective impact of stress (stress factor 1) and drug-related stress changes (factors 2 and 3) may facilitate greater emotion dysregulation, compulsive craving and drug seeking, and more-severe addiction-related distress symptoms (factor 3) in a feed-forward manner. Thus, a cumulative pattern of stress and drug misuse increases the risk of a more-chronic SUD course marked by relapses, maintenance of drug use, and treatment failure (10, 79, 136). The schematic in Figure 3 illustrates the interplay among the three stress factors that with increased levels of drug misuse and/or stress are associated with specific progressive alterations in stress- and cue-related peripheral and central adaptive stress responses, such as the prefrontal neural circuits critical to regulating peripheral, subjective, and neurocognitive control (10, 190). Other disruptions include those of the subcortical limbic-striatal circuits crucial for signaling distress, desire, and emotion, and exercising behavioral control. Together, these changes result in greater drug craving and drug intake; rigid, inflexible maladaptive coping; emotion dysregulation; and key changes in learning and memory processes that are critical for adaptive coping (10, 191).

The central GABA circuits constitute one stress processing and regulatory pathway involved in stress coping. GABA is a major inhibitory neurochemical that plays a key role in neuronal activity at the pre- and postsynaptic levels, exercising inhibitory balance and reduction of the excitatory stress arousal in hypothalamic and extrahypothalamic circuits including in the amygdala, VTA, striatal, and prefrontal neural pathways (192, 193). Notably, acute stress activates GABA simultaneously with excitatory, arousal signals, including the HPA axis; autonomic arousal responses; as well as CRF, glutamate, dopamine, and other excitatory neurochemicals involved in the cortico-striatal-limbic stress response (192–195). GABA’s complex interneuron network further aids in inhibition and modulation of stress arousal (192, 194). In this way, GABA modulates and regulates subcortical and cortical stress responses and contributes to a neural and physiologic return to homeostasis (192, 194). However, with repeated, high-intensity, and chronic stress or chronic drug exposure, GABA circuits become downregulated and dysfunctional (192, 193), which further promotes the chronic stress/drug use distress state and increased risk of stress-related illnesses such as SUD (194, 195). Whether GABA dysfunction is among the culprits facilitating elevated basal and blunted peripheral and central phasic stress responses (discussed above in “Stress, trauma, and adversity effects on addiction risk” and illustrated in Figure 1, C and D) needs further basic and clinical research. The GABA response to stress and related alterations is an example of the “double jeopardy” pathophysiology that sets in, wherein both the prefrontal-cortical circuits involved in cognitive-behavioral self-control and limbic-striatal circuits involved in signaling stress and initiating learning and motivating adaptive behavioral control are progressively disrupted by the interactive stress–drug use feed-forward cycle (shown in Figure 3) and predictive of greater drug craving, drug use, relapse, and maintenance of drug intake.

Despite the potential for heterogeneity in stress-related disruptions in SUD, it is remarkable that specific reliable stress-related disruptions are observed in clinical SUD samples and are predictive of drug craving, drug misuse, relapse, and treatment failure, as outlined in the previous sections. These biobehavioral disruptions related to SUD processes and outcomes have jointly been characterized as the “stress pathophysiology of addiction” (114), and the specific predictors in prevention, intervention, and treatment are listed in Table 2.

## Targeting stress pathophysiology in prevention, intervention, and treatment

Can the stress pathophysiology of addiction risk and relapse be targeted to restore the adaptive stress response for normal, healthy reward via social, cognitive, and behavioral coping in order to reduce drug intake and relapse and improve treatment outcomes? Research is underway to address this question, with the goal of normalizing adaptive stress response processes and improving SUD treatment outcomes. There are two specific considerations in developing interventions to target the stress pathophysiology of addiction. First, whereas there are multilevel disruptions in stress responses that encompass this pathophysiology, genetic, demographic, and clinical moderators may influence the magnitude and profile of stress pathophysiology of addiction that contribute to the significant heterogeneity discussed below. These moderators are listed in Table 3 (top) and may vary by the specific type of SUD being targeted and the specific phase of the addiction risk cycle. To address the multilevel stress disruptions in SUD, compounds or interventions that are broad-based and target the addictive processes related to stress pathophysiology of addiction are needed, such as those listed in Table 3 (bottom). These include reductions in basal and provoked stress- and cue-related drug craving; normalization of tonic and phasic changes in peripheral stress biology, including autonomic, HPA, and/or immune markers that can impact secondary SUD-related distress markers, such as sleep disturbances, fatigue, cognitive focus, and social functioning; improvements in cognitive and behavioral control and self-regulation, including anxiety, depression, and emotional reactivity; and finally, significant reductions in adverse substance use outcomes. The specific process targeted in treatment development may vary based on whether it is focused on primary prevention to reduce risk, early intervention to reduce escalation and misuse, or treatment of SUD (114).

**Figure 4:**
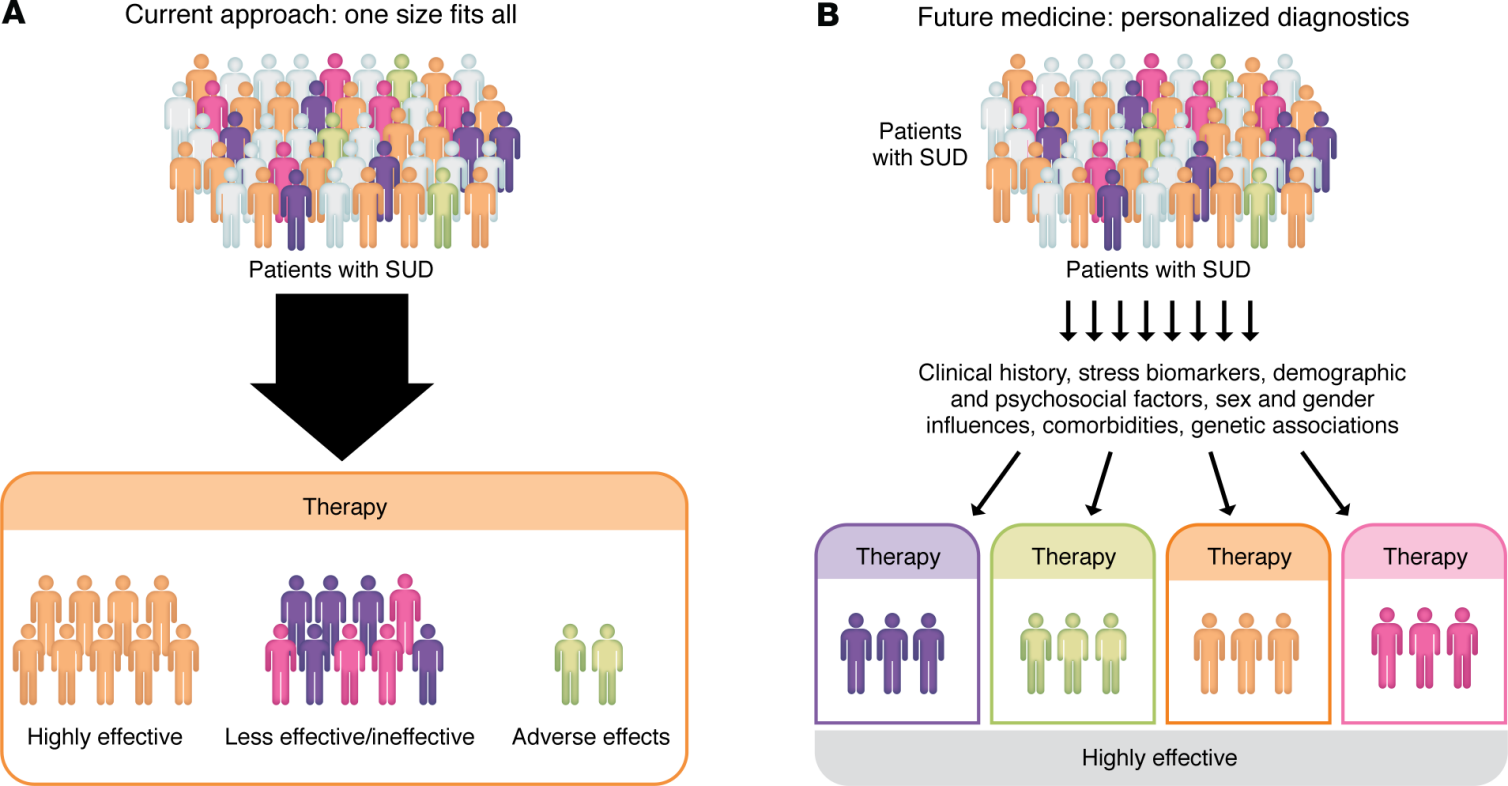
Incorporating stress response into personalized therapeutic development for SUDs. Individual differences in cumulative aggregated stress and drug misuse exposure result in substantial heterogeneity in the extent of disruption to the adaptive stress response shown in Figure 1. The general approach to intervention development is to assess therapeutics for each specific SUD, without consideration of the effects of stress and drug misuse severity levels across individuals. In the one-size-fits-all approach (**A**), all individuals are considered the same and therefore presented and treated similarly for intervention development. (**B**) Cartoon of a *precision medicine* model for a specific SUD, wherein personalized demographic, clinical, and biobehavioral markers of stress- and drug-interactive disruptions are considered as prognostic diagnostics, facilitating development of precision medicine intervention to increase SUD treatment efficacy.

How to address the heterogeneity in the stress pathophysiology of SUD? Heterogeneity is a key feature of stress-related disruptions that may vary as a function of their underlying epigenetic and molecular drivers, as discussed in several previous articles (196–198). In addition, demographic variables such as sex/gender and SUD severity, withdrawal severity, and trauma severity may also contribute to determining the magnitude and specificity of stress-related disruptions (130, 185, 199). Such variation highlights the need for precision-medicine approaches that identify subgroups of individuals based on specific moderators, such as those shown in Table 3, to examine which specific interventions, whether pharmacologic or behavioral, may improve SUD outcomes (200, 201). Notably, precision-medicine approaches, such as have been implemented in cancer treatment research (e.g., ref. 202), to identify prognostic markers and specific mediators of relapse and compulsive drug seeking in specific subgroups are needed. Adapting a similar conceptual framework for SUD, Figure 4 shows a schematic of “one-size-fits-all” intervention development versus the personalized, tailored treatment approach to address the stress pathophysiology of SUD to improve treatment outcomes.

With a focus on the broad-based stress pathophysiology markers of SUD relapse presented in Table 2, there are several examples of interventions that have shown promise in engaging the target processes outlined in Table 3. Recent evidence indicates that manipulating central glucocorticoids with mifepristone, which may normalize peripheral HPA axis responses, was useful in decreasing alcohol intake in individuals with alcohol dependence (81). Noradrenergic compounds with broad peripheral and central effects on autonomic, HPA axis, and prefrontal stress-regulatory pathways have also been examined. The α_1_-adrenergic receptor (1) antagonist prazosin reduced stress-induced alcohol craving and negative emotions, while reducing basal cortisol response and increasing stress-induced cortisol responses in inpatient individuals with AUD in early abstinence (203). This led us to hypothesize that prazosin may specifically benefit individuals with AUD in a high-distress state most broadly expressed as alcohol withdrawal symptoms. Exploring alcohol withdrawal symptoms as a clinical prognostic marker of stress pathophysiology, we found that prazosin was better than placebo in reducing alcohol use outcomes only among individuals with greater withdrawal severity but not those with AUD but no or minimal alcohol withdrawal symptoms (204). Similarly, the α_1_ antagonist doxazosin reduced cocaine use and improved abstinence outcomes in treatment-seeking individuals with cocaine use disorder (205), and some evidence also shows improved outcomes in those with comorbid PTSD and AUD (206).

Multiple α_2_ agonists have also been studied in both animals and humans to target stress-induced reinstatement of drug seeking (191). My research group found in a pilot trial of lofexidine that it reduced stress-induced opiate craving and opiate use relapse outcomes (207, 208). On the other hand, we found that guanfacine improved stress and cue-related craving and prefrontal (VmPFC) executive control function, decreased baseline cortisol response, normalized stress-induced cortisol responses, and improved drug use outcomes in SUD, but particularly in women (209–212). The guanfacine findings in SUD samples highlight the need to examine sex differences in the effects of compounds that specifically target stress pathophysiology. Indeed, some SUD medications have shown sex differences in their efficacy, including naltrexone in treating AUD (213, 214) and buproprion and varenicline in nicotine use disorder (215). Two studies with naltrexone found that men and individuals with pretreatment abstinence showed greater treatment effects, but no improvement was observed in alcohol-dependent women (213, 214). Varenicline was more effective for women compared with bupropion, while the effectiveness of bupropion was similar to that of varenicline in men (215). This research underscores the need to consider sex differences in intervention development that targets stress pathophysiology in SUD.

Finally, as a strategy to modulate endogenous GABA effects to normalize the stress pathophysiology of addiction, there has also been manipulation of sex steroids and the broad class of neuroactive steroids (NAS) in individuals with SUD. For example, chronic 5-day treatment with supraphysiologic doses of micronized progesterone (400 mg/d) versus placebo in treatment-seeking men and women with cocaine use disorder was associated with reduced cocaine craving and cortisol responses and improved prefrontal inhibitory function as measured by the Stroop task. These effects appear to be specifically related to progesterone-related increases in the GABAergic neuroactive steroid allopregnanolone (ALLO) (216, 217). Expanding on these findings, we recently showed that pregnenolone, the precursor to progesterone and other NAS, reduced stress- and drug cue–related craving and normalized basal and phasic HPA and autonomic measures of stress disruptions in individuals with AUD and cocaine use disorder (218, 219). Initial efficacy results also showed improved alcohol use outcomes (220). In summary, these examples provide initial support for intervention development to target broad-based stress pathophysiology markers in SUD with early indication of promise in improving treatment outcomes. Clearly, much more basic and clinical research is needed in this arena to assess both pharmacologic and behavioral strategies that specifically target stress pathophysiology of addiction in primary and secondary prevention, as well as SUD treatment development to improve outcomes.

## Future directions and concluding remarks

This article presents a focused review of the link among stress, trauma, and adversity and substance use, misuse, and SUD; and provides a novel adaptive stress response conceptual framework to understand the stress-related dysfunctions associated with addiction risk and in SUD. A multilevel dynamic, flexible, and adaptive stress response is described to illustrate changes in the responses that occur with stress, trauma and adversity, drug use and misuse, and postdependent abstinence- and withdrawal-related stress in the pathophysiology of addiction. Such stress disruptions have been associated with increased drug craving and compulsive drug intake and risk of relapse and treatment failure. These findings support the premise that broad-based interventions are needed that can reverse and rescue the stress disruptions in addiction risk and normalize the flexible, adaptive stress response while improving substance use outcomes in SUD treatment. This requires expanded basic research with novel approaches to capture multilevel stress responses in animal models. For example, peripheral autonomic and HPA axis changes associated with chronic stress and chronic drug use that contribute to limbic striatal adaptations in molecular pathways may help identify specific mechanisms driving multilevel adaptations to the stress response and its related behavioral sequelae. Such research could identify new molecular drivers of the multilevel stress responses that could lead to novel treatment targets to break the stress-drug misuse cycle and also improve substance misuse outcomes. Thus, basic and clinical research aimed at understanding more fully the stress pathophysiology of addiction and developing novel behavioral, social, and pharmacologic interventions to address this pathophysiology is needed to prevent and treat SUD. Such developments would profoundly benefit affected individuals by reducing SUD-related morbidities and prevent the development of SUD by reducing addiction risk.

## Figures and Tables

**Table 3:**
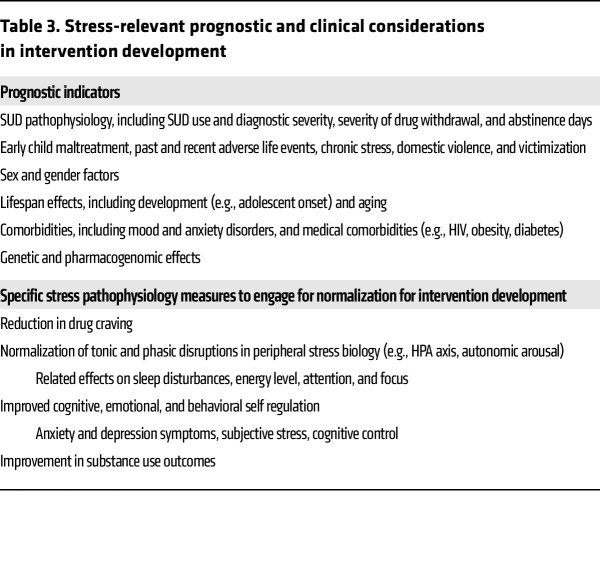
Stress-relevant prognostic and clinical considerations in intervention development

**Table 2:**
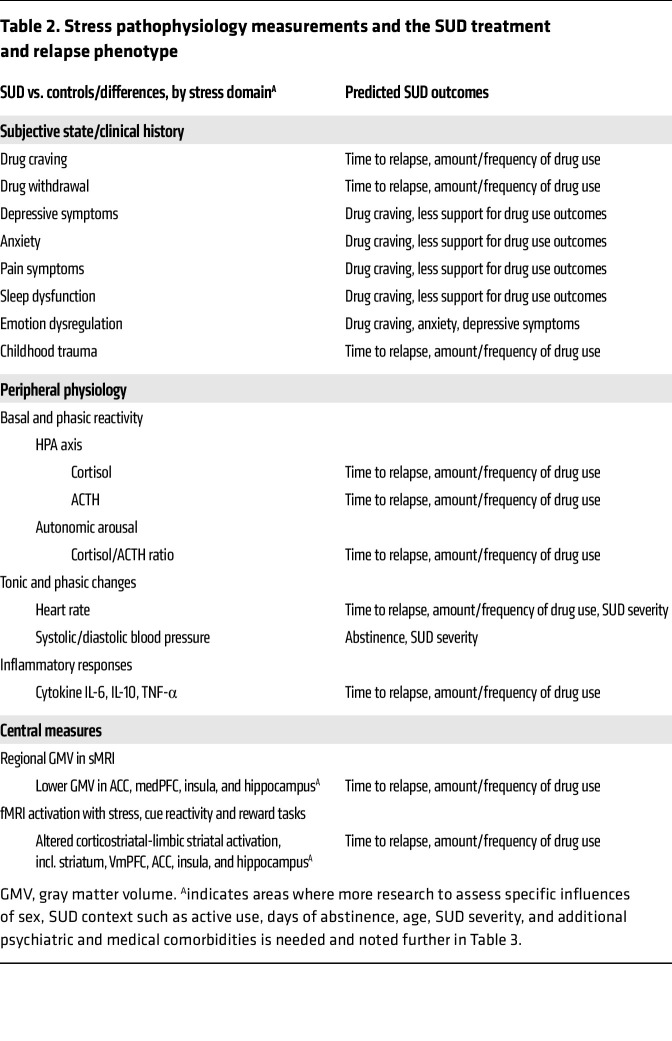
Stress pathophysiology measurements and the SUD treatment and relapse phenotype

**Table 1:**
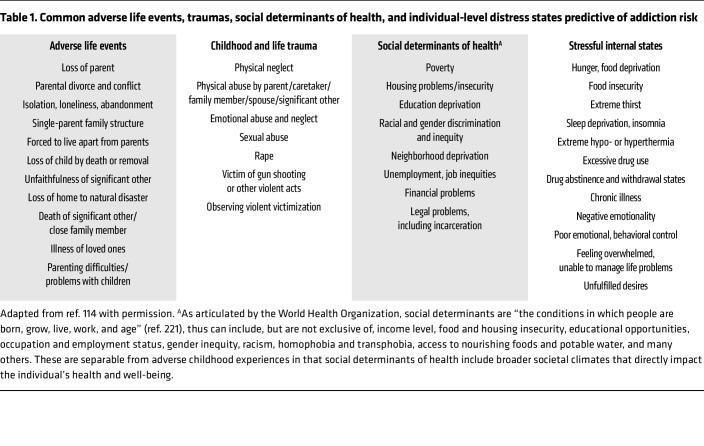
Common adverse life events, traumas, social determinants of health, and individual-level distress states predictive of addiction risk

## Version 1 08/15/2024

Electronic publication
